# Organizing an Orthopaedic Department During COVID-19 Pandemic to Mitigate In-Hospital Transmission: Experience From Greece

**DOI:** 10.7759/cureus.8676

**Published:** 2020-06-17

**Authors:** Eustathios Kenanidis, Panagiotis Anagnostis, Kostoula Arvaniti, Michael E Potoupnis, Eleftherios Tsiridis

**Affiliations:** 1 Academic Orthopaedic Department, Papageorgiou General Hospital, Aristotle University Medical School, Thessaloniki, GRC; 2 Center of Orthopaedic and Regenerative Medicine - Center of Interdisciplinary Research and Innovation, Aristotle University Medical School, Thessaloniki, GRC; 3 Academic Orthopaedic Department, Papageorgiou General Hospital, Thessaloniki, GRC; 4 Critical Care, Papageorgiou General Hospital, Thessaloniki, GRC; 5 Academic Orthopaedics, Papageorgiou General Hospital, Thessaloniki, GRC

**Keywords:** covid-19, novel coronavirus, sars-cov-2, pandemic, orthopaedic department, operating theatre, hospital admission

## Abstract

The new severe acute respiratory syndrome coronavirus 2 (SARS-CoV-2) emerging in Wuhan city of China, was the cause of a rare type of pneumonia evolving rapidly in pandemic early at the beginning of 2020. The rapid human-to-human transmission of SARS-CoV-2 increases the risk of in-hospital transmission, requiring re-definement of musculoskeletal trauma management and postoperative care. Following the review of the existing literature on COVID-19 and similar infectious diseases, National and Hospital Board instructions for Infectious Diseases, as well as the consensus for surgical care by the consortium of the Orthopaedic Department Directors, we present the outline of the implemented principles in the orthopaedic departments of a tertiary academic hospital in Greece to operate during COVID-19 pandemic. Our overall objectives were to decrease the admission load and mitigate the risk of in-hospital transmission of SARS-CoV-2. The principles involve the management of the Orthopaedic medical and nursing personnel, alterations of the workflow in the wards, operating rooms and outpatient clinics from the admission to the discharge of an orthopaedic patient. In addition, we present the recommended principles of management of traumatic orthopaedic injuries highlighting those deserving admission and in-hospital care and those that can be treated in the outpatient setting or day surgery clinics.

## Introduction and background

The new severe acute respiratory syndrome coronavirus 2 (SARS-CoV-2) has arisen as the reason for an uncommon group of viral pneumonia cases in China, evolving rapidly into a worldwide health emergency [1]. On 12 March 2020, the World Health Organization (WHO) announced coronavirus disease (COVID-19) outbreak a pandemic causing international concern and global anxiety [2]. At the time of writing this article, the number of confirmed cases worldwide has exceeded 2,400,000 [3]. As the rapid human-to-human transmission of SARS-CoV-2 has been established, the risk of in-hospital transmission is high [4]. The spread of the virus is expected following the care of the confirmed COVID-19 patients but may be equal for symptomatic and asymptomatic patients that are infected [5]. 

The government of Greece has taken strict hospital and social distancing measures early at the very beginning of the COVID-19 outbreak reducing transmission and limiting the need for in-hospital or intensive unit care. At the time of writing, we had 2401 confirmed positive patients, of which 121 died [3]. The exposure and immunity of the population are largely unknown as only 0.57% of the population of Greece has been tested [6]. The spread of SARS-CoV-2 in the community remains unknown; thus, the mitigation of in-hospital transmission remains a considerable task. Overall, Greece's performance has been satisfactory in mitigating SARS-CoV-2 transmission and infection, thereby limiting morbidity and mortality. Thus far, the Greek National Health Service has successfully been able to accommodate the demand for inpatient care during the pandemic [6].

The aim of this paper is to review the existing orthopaedic literature and to present the principles of management and care implemented in the orthopaedic departments of a tertiary academic hospital in Greece to operate during COVID-19 pandemic in order to mitigate the risk of in-hospital transmission of SARS-CoV-2 to the medical, nursing and administrative orthopaedic personnel. The principles included the management of the staff and the modification of workflow and practices in the wards, the operating rooms and the outpatient clinics beginning from the event of admission and up to the discharge of an orthopaedic patient. In addition, we presented the clinical indications to delineate orthopaedic patients who deserve emergency or urgent in-hospital care from those that can be treated in the outpatient setting, as well as from the day surgery clinics or could not be admitted in the hospital, in order to decrease the SARS-CoV-2 transmission load. These principles are based on the existing orthopaedic literature on COVID-19 prevention and care as well as similar infectious diseases from the past, the Greek National Society of Public Health and Infectious Diseases, the Hospital Board of Infectious Diseases, the Hospital Medical Management Board and the consensus for surgical care by the consortium of the Orthopaedic Department Directors of our tertiary academic hospital [6-8]. 

## Review

The proposed principles of management and care are deployed below as (1) general management of the orthopaedic departments, (2) recommendations for the management of traumatic orthopaedic injuries, (3) hospital pathways for the admitted orthopaedic patients (4) workflow of the isolated and negative pressure COVID-19 operating theatre (COT) and (5) postoperative care of the COVID-19 infected patients.

General management of the orthopaedic department

Orthopaedic Patients Admission into Three Distinct Clinical Areas

**Figure 1:**
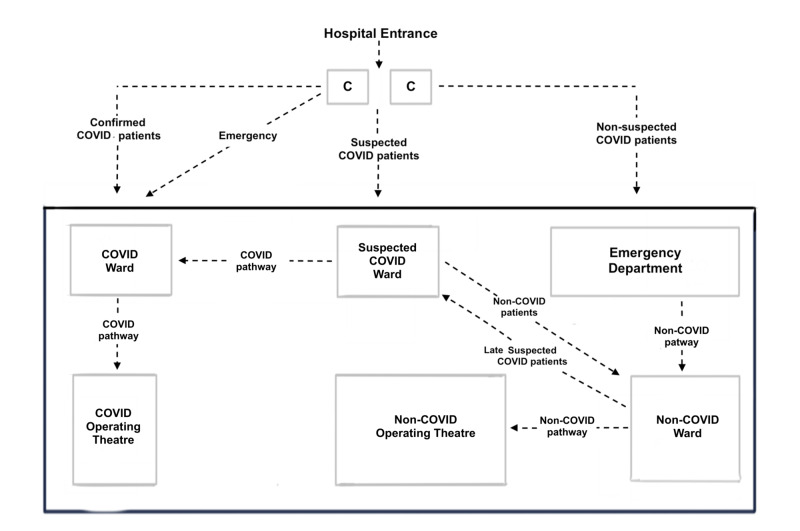
Hospital pathways for the admitted patients (1) COVID non-suspected: Emergency Department > Non-COVID ward > Non-COVID operating theatre (2) COVID confirmed: COVID ward > COVID operating theatre (3) COVID suspected: suspected COVID ward > split either COVID or Non-COVID ward > COVID or Non-COVID Operating Theatre (4) Emergency: no time for COVID triage; consider and treat as COVID confirmed C: cubicles ahead of the emergency department, outside the hospital for COVID triage of patients

Νon-COVID-19 patients must be admitted in the standard non-COVID Orthopaedic (NCO) wards and the COVID-19 patients in the special negative pressure isolated COVID Orthopaedic (CO) wards [9]. The suspected but unconfirmed COVID-19 patients who need orthopaedic care should temporarily be admitted in a dedicated ward for suspected patients (SCO) away, and separated from CO wards until the molecular testing for COVID-19 becomes available (Figure 1). 

The results of the tests were available within 24 hours. Infectious disease specialists performed the screening in the cubicles just in front of the entrance of the hospital (Figure 1). The screening was based mainly on the following criteria: the previous testing for COVID-19, living with a person that was affected of COVID-19 or was a health worker, travelling abroad, contact with a suspected person, smoking, symptoms mainly fever>38^0^, chest pain, trouble breathing, fatigue, chronic conditions as asthma and other pulmonary chronic conditions, chronic kidney disease, cancer and diabetes.

Decreased Admission Rate 

All elective orthopaedic surgery cases were postponed [10]. Only oncological cases following the oncology MDT (multidisciplinary oncology team) evaluation and life or limb-threatening injuries, were admitted for surgery [11-12]. Due to the reduction of elective admissions, NCO orthopaedic wards were amalgamated in order to free staff and beds for CO wards.

Non-COVID patients suffering from non-life- or limb-threatening injuries were admitted in the NCO wards if surgery was to be performed urgently; patients who could clinically afford to be operated on at a later stage were discharged home, with a specific plan to be admitted for day-case surgery when indications and operating room availability allowed [13]. Non-COVID patients admitted for surgery were discharged postoperatively as soon as possible without compromising their health. Patients who could be treated conservatively were discharged from the emergency department (ED) and followed up in the outpatient clinic with a specific appointment in a dedicated outpatients area [13].

COVID patients should be admitted in the CO wards and preferably treated conservatively or at a later stage if possible. If they needed urgent surgery, management was discussed by a multidisciplinary team comprising of the anesthesiologist, the orthopaedic surgeon, the infectious disease internist. The elected procedure was performed in the specially dedicated negative pressure COVID operating theatres (COT) under the management of a special COT nurse coordinator. 

NCO Wards Personnel Management 

The staff was advised to avoid needless travelling. The temperature and respiratory symptoms of all staff were monitored by the head of the nursing staff, twice a day in the beginning and the end of the shift to identify staff at risk [9, 12]. 

Pregnant, immunocompromised or over 60 years of age staff were protected; thus, we preferred to involve healthy younger personnel for the care of COVID-19 patients [12]. In addition, personnel working at the CO wards were not mixed with personnel at NCO wards at any time [12]. Standard staff groups comprising of designated consultants-residents-nurses were put on duty together; thus, if one of the group members was infected, only the group was quarantined and not the whole department. As the Greek Government imposed strict social distancing measures, and the incidence of musculoskeletal injuries and subsequent admissions was lessened by 50%, the number of medical and nursing staff required during the on-call duty was also reduced. During the trauma on-call, we involved two 12 hours shifts, each one of which comprised of two residents in the ED, two residents in the NCO wards and two consultants. All medical staff received special training on how to properly dress, enter and exit the CO wards and COT; the nursing staff was is standard shifts in CO wards and COT wearing personal protective equipment (PPE) at all times. The catchment area of our Hospital is 1.5 million people populated urban area. 

Orthopaedic Staff Education 

All staff required provision of PPE (N95 respirators, personal goggles, gown and gloves) and were trained systematically to use PPE, especially cleaning, disinfecting, storing, and inspecting PPE for any damage [12]. The use of a simple surgical mask was advised for the routine care of low-risk patients (no fever or respiratory symptoms, no history of recent travel or close contact with a COVID-19 patient) [9, 12]. Once the community spread of the virus was confirmed, routine prevention measures included the wearing of N95 masks, eye protection, gown, and gloves [7, 12, 14]. 

NCO Wards Workflow Modifications 

The initial patient clinical screening in the ED (medical history, clinical examination, temperature) could not thoroughly exclude the possibility of infection, due to the 14 day long incubation time of SARS-CoV-2 [1, 4]. Following admission, fever, respiratory and gastrointestinal symptoms, as well as the loss of smell and taste or myocardial dysfunction, were monitored every day to identify late COVID-19 patients [1, 4, 9]. Suspected inpatients were immediately put to the deep pharyngeal swab Polymerase Chain Reaction (PCR) testing for SARS-CoV-2, and the same time moved to the designated SCO wards until the result was made available. Transferring the suspected patient was challenging; with the patient always wearing a full face mask and via a specific pathway when possible within the hospital. Contamination of the NCO wards from a late COVID-19 positive case would require NCO ward closure, disinfection and 14-day quarantine of the involved personnel.

Recommended management of orthopaedic trauma

The management of orthopaedic trauma during the pandemic was modified to mitigate the risk of SARS-CoV-2 in-hospital transmission. Admission was offered to patients suffering from life or limb-threatening injuries needing emergent or urgent surgery [13]. All other patients needing surgery were admitted only if the urgent procedure could be planned; otherwise, patients were discharged and scheduled for day surgery at a different timepoint [13]. All other injuries were not admitted but managed in the ED and followed up in the outpatient clinics. All patients admitted for surgery should undergo a deep pharyngeal swab PCR testing for SARS-CoV-2 (Table 1). 

**Table 1:** Recommended management of traumatic injuries during COVID-19 pandemic ^1^ Non-absolute indication for admission (non-life- and limb-threatening injuries); admit if urgent surgery is planned otherwise discharge and plan for day surgery ^2^ No indication for admission: to be managed in the ED and followed in the outpatient clinic ^3^ To facilitate prompt treatment, especially in the absence of qualified surgeons, hemiarthroplasty may be the best treatment option for the majority of patients with sub-capital hip fractures

Absolute Indication for admission	Relative Indication for admission^1^	Contraindication to admission^2^
Polytrauma patients	Peri-articular fractures	Stable upper/ lower limb fractures +/- minimal displacement
Pelvic & acetabular fractures	Intra-articular fractures	Stable spinal fractures
Spinal fractures with instability and /or neurologic impairment	Lower limb: knee, tibial, foot & ankle injuries	Dislocations of native and replaced joints with acceptable reductions
Pathological fractures due to primary/metastatic bone disease	Upper limb injuries needing surgery as forearm, wrist or proximal humeral fractures	Upper limb fractures (clavicle, humeral, wrist) with high union rates
Musculoskeletal injuries with associated vascular or neurological morbidity	Non-unions which threaten the soft-tissue envelope	Non-contaminated penetrating limb injuries with no neurological or vascular deficit
Prosthetic joint infections or infected fracture fixation with life-threatening uncontrollable sepsis	Ligamentous injuries of the knee and the elbow requiring operative treatment	Abscesses in patients without systemic sepsis
Septic arthritis/ systemic sepsis		
Cauda equina syndrome		
Compartment syndromes		
Open fractures		
Hip fractures^3^		
Femoral fractures		
Irreducible joint dislocation		

Hospital pathways for orthopaedic patients 

Two special cubicles were located in the area in front of the ED and outside the hospital for COVID-19 triage. No patient could enter the ED or the outpatient clinics without entering the COVID triage first. COVID-19 non-suspected, suspected or confirmed patients were managed in dedicated areas and wards via specific pathways, as indicated in Figure 1. The suspected COVID-19 patients were managed in the SCO area, where a chest radiograph and a deep pharyngeal swab PCR testing for SARS-CoV-2 were taken. When admission was deemed necessary, patients remained in the SCO ward until the test result was made available; in all other cases, the patient was advised to return home for isolation, waiting for the result. Emergency trauma or polytrauma patients were treated as COVID-19 suspected cases regardless of their symptoms and until proven otherwise and followed the above screening process in the SCO area (Figure 1).

COVID operating theatre (COT) workflow

Designation of the COT 

Two operating theatre complexes with negative pressure and ventilation systems and an integrated high-efficiency particulate air (HEPA) filter to decrease viral dissemination were chosen for surgery of COVID-19 patients [7, 9, 12, 15]. The COT was autonomous, immediately next to the CO wards and non-connected with NCOT to diminish the risk of contamination of non-COVID patients [12]. The COVID triage, CO ward and COT were all distinct, isolated and marked with yellow floor tape and named COVID pathway [12] (Figure 1).

Organising the COT 

Experienced and limited numbers of nursing and medical personnel were dedicated to the surgical care of COVID-19 patients in every shift [12, 16]. The majority of them who were senior enough in order to execute quick and efficient surgery were all provided with the necessary PPE [16]. Preoperatively surgeons, anaesthesiologists and scrub nurses should meet to ensure proper coordination and surgical planning [12]. In addition, a senior staff nurse was coordinating the staff, making sure that communication and limited COT traffic was occurring during surgery [12].

All equipment and drugs needed for the surgery should be preselected and brought into the COT. In addition, all anaesthetic monitors, computers, and ultrasound device surfaces should be covered with plastic wrap to decrease the risk of contamination and to facilitate cleaning [12]. Whenever possible, single-use equipment could be used [17-18]. New workflows of staff, senior coordination, movement of medical equipment, infection prevention practices, and decontamination were established following the procedure [12]. 

Anaesthesia of the COVID-19 Patients

The most experienced anaesthesiologist using a powered air-purifying respirator (PAPR) ought to perform the anaesthesia for speed and accuracy [19]. All anaesthesia has to be induced in the COT directly, and no preoperative anaesthetic room allowed to be used. Regional is favoured over general anaesthesia with the patient wearing a surgical face mask during the operation [12, 16]. When needed, oxygen is administered via nasal prongs under the surgical mask [9, 19].

Aerosol-generating procedures (airway manipulation, face mask ventilation) should be avoided to reduce the risk of viral aerosolization [12, 17, 19]. When general anaesthesia is decided, pre-oxygenation could be performed via a well-fitting face mask. Bag-mask ventilation is not recommended, but if unavoidable, one should proceed with small tidal volumes at low pressure [19]. Before intubation, deep anaesthesia is advised [19]. Video-laryngoscopy for intubation is highly recommended as the anaesthetist stays away from the patient’s airway and PPEs usually impede vision during direct laryngoscopy [9]. During intubation, a closed airway suctioning with a rigid suction catheter to decrease the chance of infecting the surroundings could be an alternative procedure [17]. It is recommended to use anti-emetics to diminish the risk of postoperative vomiting [12]. After extubating, the patient must wear a surgical face mask, and nasal prongs underneath the face mask to receive supplemental oxygen [17, 20]. The patient should fully recover in the COT and be transferred directly to the CO ward as recovery areas are not available for safety reasons.

Managing Orthopaedic Surgery for COVID-19 Patients

Only oncological and emergency life or limb-threatening orthopaedic trauma surgeries were allowed for COVID-19 confirmed patients [12-13]. Blood donations are limited during COVID-19 pandemic; thus, adequate blood inventory preoperatively is necessary to support the procedure [21-22]. Preoperative templating is required to choose the appropriate type of implant and to decrease intraoperative exposure and time. COVID-19 patients should wear a surgical face mask at all time [12, 14].

The most experienced surgeon with limited scrubbed personnel using well-fitted N95 respirator, eye protection, cap, gown, and double gloves, should perform the operation in a fast and efficacious manner [12]. Damage control surgery principles should apply for emergency and urgent orthopaedic surgery. When possible closed reduction and K-wires or external fixators should be elected to limit exposure and surgical time [13]. Smoke suction diathermy must be used to eliminate spread and contamination, and absorbable sutures were advised for closure [13]. Full documentation of all involved staff is in place to facilitate contact tracing. All staff should also take a shower and change into a clean set of scrubs following surgery [12].

Postoperative care of COVID-19 infected patients 

In Hospital

Routine postoperative ward rounds should be minimized to reduce movement of staff, exposure and spread of COVID-19 into the hospital [12]. The transportation of infected patients is also limited; when possible, portable x-ray equipment should be used for postoperative diagnostic imaging [23-24]. If this is not possible, a satellite radiography centre using dedicated radiography equipment is developed next to the CO ward to decrease the risk of transmission [24]. If the transfer of a patient to the regular radiology department is unavoidable, the patient should wear a surgical mask during the transfer [23]. All radiological equipment, including probes and image viewing station mouse and keyboards, need to be disinfected after every contact with suspected or infected patients [23-24].

Outpatient Follow-up

Postoperative rehabilitation may not be feasible. As a result, early postoperative full weight-bearing of patients operated on for hip fractures should allow early rehabilitation and discharge [13]. Easily changeable postoperative dressings and splints would be selected to facilitate remote follow-up [13]. Follow-up appointments are minimised to those necessary; a postoperative x-ray is asked when it is possible to make an important adjustment in management. Remote follow-up using telephone or video calls are also considered [13]. Postoperative imaging is also decreased to those necessary; however, a postoperative x-ray is asked when it is possible in order to make an important adjustment of management [13].

## Conclusions

In the era of COVID-19 pandemic, the knowledge and the information applied to the clinical practice in orthopaedics change daily. The basic principles that govern the musculoskeletal hospital care are re-designed to build a universal model for the National Health Services. The orthopaedic setting of a hospital is split into two pathways, the COVID and non-COVID. These pathways are distinct, far away from each other, separately equipped, and separately staffed with properly trained nursing personnel. 

In our paper, we provide a process of care for the admitted, emergency and urgent musculoskeletal injuries; the majority of the orthopaedic cases are either postponed or treated conservatively. In the future, we will have the opportunity to evaluate the outcome and the morbidity caused by this management. 
